# The PHA Test Reflects Acquired T-Cell Mediated Immunocompetence in Birds

**DOI:** 10.1371/journal.pone.0003295

**Published:** 2008-09-29

**Authors:** José L. Tella, Jesús A. Lemus, Martina Carrete, Guillermo Blanco

**Affiliations:** 1 Department of Conservation Biology, Estación Biológica de Doñana, Consejo Superior de Investigaciones Científicas, Sevilla, Spain; 2 Departament of Evolutive Ecology, Museo Nacional de Ciencias Naturales-CSIC, Madrid, Spain; New York University School of Medicine, United States of America

## Abstract

**Background:**

cological immunology requires techniques to reliably measure immunocompetence in wild vertebrates. The PHA-skin test, involving subcutaneous injection of a mitogen (phytohemagglutinin, PHA) and measurement of subsequent swelling as a surrogate of T-cell mediated immunocompetence, has been the test of choice due to its practicality and ease of use in the field. However, mechanisms involved in local immunological and inflammatory processes provoked by PHA are poorly known, and its use and interpretation as an acquired immune response is currently debated.

**Methodology:**

Here, we present experimental work using a variety of parrot species, to ascertain whether PHA exposure produces larger secondary than primary responses as expected if the test reflects acquired immunocompetence. Moreover, we simultaneously quantified T-lymphocyte subsets (CD4^+^, CD5^+^ and CD8^+^) and plasma proteins circulating in the bloodstream, potentially involved in the immunological and inflammatory processes, through flow cytometry and electrophoresis.

**Principal Findings:**

Our results showed stronger responses after a second PHA injection, independent of species, time elapsed and changes in body mass of birds between first and second injections, thus supporting the adaptive nature of this immune response. Furthermore, the concomitant changes in the plasma concentrations of T-lymphocyte subsets and globulins indicate a causal link between the activation of the T-cell mediated immune system and local tissue swelling.

**Conclusions/Significance:**

These findings justify the widespread use of the PHA-skin test as a reliable evaluator of acquired T-cell mediated immunocompetence in diverse biological disciplines. Further experimental research should be aimed at evaluating the relative role of innate immunocompetence in wild conditions, where the access to dietary proteins varies more than in captivity, and to ascertain how PHA responses relate to particular host-parasite interactions.

## Introduction

Immunocompetence has reached a central focus in evolutionary and behavioural ecology after the general upsurge of interest in host-parasite interactions, to the point of the emergence of immunoecology as a new scientific discipline [1]. Birds have been used as the main models for testing a variety of hypotheses and life-history trade-offs, for which researchers have been prompted to learn and apply immune techniques suitable for both laboratory and field experiments. The PHA-induced skin swelling test has been intensively used, and is now considered a classical immunological technique [2]. Adapted from the poultry science methods used in the seventies [3], the technique consists of subcutaneous injection of the mitogen phytohemagglutinin (PHA) dissolved in phosphate-buffered saline (PBS), usually in the wing patagium, and quantifying concomitant swelling at the site of injection over time. The resulting swelling, usually measured 24 h post-injection, is interpreted as an index of cell-mediated immunocompetence [2], [4]. The extreme popularity of this immune test seems to arise from its simplicity, requiring little training and no laboratory facilities, and its feasibility *in vivo* under field conditions [2]. Reflecting its wide use, the simplified protocol proposed by Smits et al [4], which involves avoiding the injection of PBS in the opposite patagium as an unneeded control [2], has been cited ca. 200 times since its publication in 1999 (ISI web of Science, acceded on August 2008). The test has broadened its applicability not only in birds but also in fishes, amphibians, reptiles and mammals [5]–[8] to address a variety of questions in recent years, covering a range of topics from classical host-parasite interactions [7], [9] to the evolution of coloration [5], [10], behaviour [11], [12], mating systems [13], physiological trade-offs [10], [14], immunocompetence [15], [16], foraging strategies [17], ecotoxicology [18], [19], veterinary sciences [20] and conservation biology [6], [21].

The PHA test has been used as a measure of T-cell mediated immunocompetence after the pioneering work by Goto *et al* [22], who showed a reduction of the skin response in thymectomized chickens (thus being unable to produce circulating T-cells). Doubts have arisen in recent years, however, among evolutionary ecologists about the true nature of the immune reaction provoked by PHA and its interpretation. Recent histological work has shown the intensive infiltration of many immune cell types in the PHA-injected patagium of birds over the course of the swelling response, involving both innate and adaptive components of the immune system, and cautions against interpreting larger swellings as greater cell-mediated immunocompetence [2]. Since B and T lymphocytes were not distinguishable in that histological study, there is still a need to differentiate between innate, nonspecific inflammatory reactions and the acquired, or specific cell mediated immune responses supposedly tested through PHA injection. Moreover, there is no conclusive evidence supporting secondary responses as greater after a previous exposure to PHA, as expected from a true acquired immune response [23]. Overall, the use of the PHA test as a surrogate of T-cell mediated immunocompetence has been recently questioned, posing serious doubts about the interpretation of a huge amount of previous work based on this technique [23]. Here, we present experimental results showing that secondary responses to PHA injection are consistently larger than primary ones in a number of bird species. Moreover, the simultaneous quantification of several T-lymphocyte subsets and proteins circulating in the bloodstream (see below) shows that PHA-induced skin swellings are related to the proliferation of T-cell subsets responsible for the acquired immune response, thus supporting this test as a reliable technique to measure *in vivo* T-cell mediated immune responses.

### Components of the immune response

We measured blood-circulating T lymphocytes and proteins associated with the cellular and innate immune responses supposedly elicited by the PHA-immune challenge. Circulating T lymphocytes produced in the thymus, which are characterised by their expression of special T cell receptors (TCR), are responsible for the cell-mediated immune response in vertebrates. Briefly, T-cells are a group of very distinct subsets among which the most abundant are CD4^+^ (active lineages), CD5^+^ (adyuvant lineages), and CD8^+^ cells (memory or antigen presenting cells). The first two subsets are implicated in cellular based defence, while CD8^+^ constitute the most common memory subset. The CD4^+^ subset is implicated in the production of several active substances, such as cytokines, interferon and several types of interleukin, such as interleukin -6 (IL-6). The CD4^+^ subset participates in the first phase of the skin swelling response (i.e., 6–12 h after injection), where there is exudation of plasma from surrounding vascular tissues and edema at the injected site, by activating local innate cell populations (mainly basophils and macrophages) [24]. The CD5^+^ subset is far less common and more specialised, being implicated in intracellular kinase activity (activator and substrate) and cell mediated signalling as well the maturation of other T-cell subsets [25]. The CD8^+^ subset, on the other hand, is responsible for specific antigen expression, although it also presents discrete cytolytic activity [26].

Albumin is the largest single fraction of circulating protein in healthy birds. It serves as the major protein reservoir, the main contributor of colloidal osmotic pressure, a participant in acid-base homeostasis, and transport carrier for small molecules such as minerals, hormones, vitamins and fatty acids. Albumin is transformed by the organism into globulins when facing an infection process [27]. Alpha globulins (α-globulins) are a very heterogeneous group of proteins manufactured almost entirely by the liver, including many transport proteins such as lipoproteins (α-1), haptoglobin, ceruloplasmin, and macroglobulins (α-2) [28], [29]. Because many of these α-globulins function as acute phase proteins which are elevated in inflammatory processes, they serve as a useful index in the diagnosis and monitoring of many infectious diseases and other causes of acute or chronic inflammation [28]. Briefly, α-1 globulins (lipoproteins) transport lipids throughout circulation, while among α-2 globulins, haptoglobin protects kidneys from tissue destruction by binding free hemoglobin after hemolysis. Ceruloplasmin, an antioxidant glycoprotein, transports copper to cells while macroglobulin is unique as an antiproteinase both in terms of the broad spectrum of enzymes that it can inhibit and the nature of its inhibitory activity. One or two of these subfractions can be identified by electrophoresis [30]. Betaglobulins (β–globulins) are also heterogeneous, including C-reactive protein, complement, and fibrinogen (β-1), or carrier proteins such as lipoproteins and transferrine- (β-2). Many of the β-globulins are also acute phase proteins. Among β-1 globulins, complement is the primary mediator of the antigen-antibody reaction. The C reactive protein plays an important role in initiating and modulating inflammatory and immune responses while fibrinogen plays an important role in homeostasis, but is also the main contributor to inflammatory and tissue repair processes. β-2 globulins, on the other hand, transport lipids throughout circulation (high molecular weight lipoproteins) and carry iron from the cells involved with the absorption or storage of iron (transferrin). Contrary to mammals, no immunoglobulins are present in this fraction in birds [27]. As in the α-globulins, one or two subfractions can be identified [30]. Finally, the gamma (γ-globulins) fraction contains most of the immunoglobulins in birds, which are involved in the humoral immune response.

## Results and Discussion

**Figure 1:**
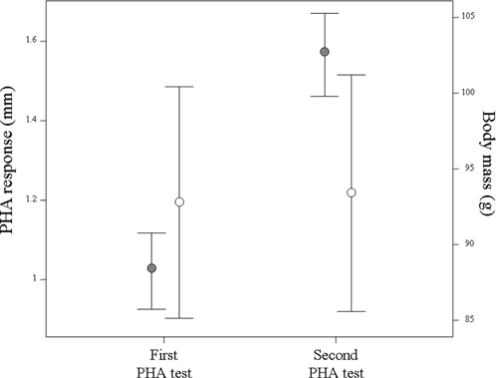
Local tissue swelling (black dots) and body mass (white dots) of 125 birds subjected to two successive PHA-skin tests.

The PHA-induced tissue swelling was on average 86% larger in the second than in the first immune challenge (time range between PHA challenges: 5–250 days), with only four out of 125 individuals showing small increases (<10%). Changes in body mass, however, were unappreciable (0.43% on average) between the first and second immune tests (Fig. 1). The second PHA-induced tissue swelling was significantly larger than the first (F_1,123_ = 482.67, P<0.0001), while controlling for the species body mass (F_1,123_ = 26.94, P<0.0001) and the significant differences in PHA responses among the variety of species tested (Z = 7.22, P<0.0001). The swelling increase of individuals (i.e., the absolute difference between the second and the first response) was unrelated to their changes in body mass (*F*_1,83_ = 0.02, *P* = 0.89) or time elapsed between the two immune tests (F_1,83_ = 2.6, P = 0.11), while controlling again for body mass (F_1,83_ = 1.02, P = 0.31) and species (Z = 0.67, *P* = 0.25). To our knowledge, this is the first experimental work designed to test larger secondary than primary responses to PHA injection. Nonetheless, the literature shows anecdotal and inconsistent evidence obtained from unrelated experiments and small sample sizes: four captive birds used as experimental controls showed larger secondary responses [31], while four wild birds recaptured and tested for immunity about one year later [32] and ten captive birds resampled after four months [33] did not show appreciable differences between the first and the second immune challenge. A better sample consisting of 86 broods of nestling passerines showed larger secondary swellings when challenged two days after a first injection, results which were not interpreted as evidence of acquired immunity but rather as a matter of methodology to be considered in statistical analyses [34]. These larger secondary responses, however, could be confounded by ontogeny and thus reflect to some extent the development of immunocompetence during the growth period of chicks in the nest [35]. Our results, however, clearly demonstrate that the PHA-test provokes a greater swelling on the second compared with the first PHA response, a critical requirement if the test is to be considered indicative of a true cell-mediated acquired immunity [23]. Moreover, greater secondary responses appear as soon as five days after the first PHA injection as well as long time after injection (at least 250 days), and thus could provide lifelong immunity upon subsequent exposure as expected from the acquired T-cell mediated immune system [23].

**Figure 2:**
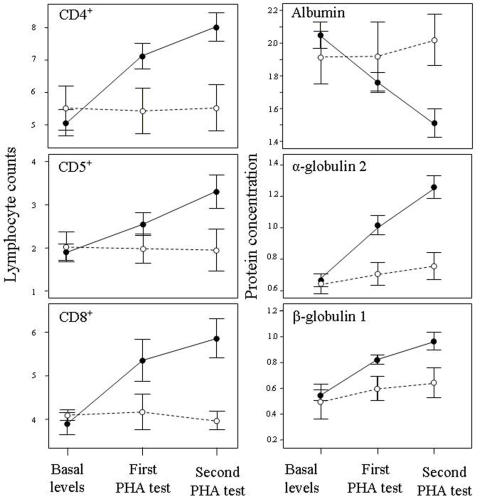
Changes in counts of lymphocyte subsets and protein concentrations (μg/L) in the bloodstream from basal levels to those resulting 24 h after a first and a second PHA injection. Black dots represent experimental birds (injected with PHA) and white dots represent control birds (injected with PBS).

A concomitant prediction is that if the swelling is produced by the proliferation of T lymphocytes typically involved in the acquired immune response, then there is likely to be a much lower level of these lymphocytes at first exposure compared to subsequent exposures to PHA [23]. Our flow cytometry results fully support this. The increases in circulating T-lymphocyte subsets from basal levels to profiles resulting after the first and second PHA-tests (Fig. 2) were significant for both CD4^+^ active subset (F_2,28_ = 290.57, P<0.0001), CD5^+^ coadyuvant subset (F_2,28_ = 82.74, P<0.0001) and CD8^+^ memory subset (F_2,28_ = 182.33, P<0.0001), while controlling for significant differences among the species tested (all P<0.006). There were always significant differences between the three instances of blood sampling (Helmert transformation, all P<0.0001). As expected, control birds (injected only with PBS) did not show changes in lymphocyte profiles (P-range: 0.38–0.85, Fig. 2). Therefore, PHA exposure is clearly responsible for the proliferation of lymphocytes associated with an acquired T-cell mediated immune response.

Plasma protein concentrations of experimental birds also changed as a response to both first and second PHA injections (Fig. 2). There was a significant decrease in albumin (F_2,28_ = 71.75, P<0.0001) at the same time that α-globulin 2 (F_2,28_ = 201.88, P<0.0001) and β-globulin 1 (F_2,28_ = 76.22, P<0.0001) increased as a consequence of their contribution to the inflammatory process (Fig. 2), while controlling for significant differences among species (all P<0.05). In turn, production of α-globulin 1 and β-globulin 2 were increasingly inhibited during the experiment, as indicated by their decreasing concentrations (F_2,28_ = 311.53, P<0.0001 and F_2,28_ = 156.54, P<0.0001, respectively; not shown in Figure). There were always significant differences between the three temporal series of blood samples (Helmert transformation, all P<0.004). However, there was minimal change in the protein profiles of control birds (P-range: 0.05–0.67 for α = 0.01 after Bonferroni correction; Fig. 2). As expected, γ-globulins, which are involved in the humoral but not in the cellular immune response, did not change after PHA injections (F_2,28_ = 2.45, P = 0.10, species effect: F_4,14_ = 1.51, P = 0.25).

**Figure 3:**
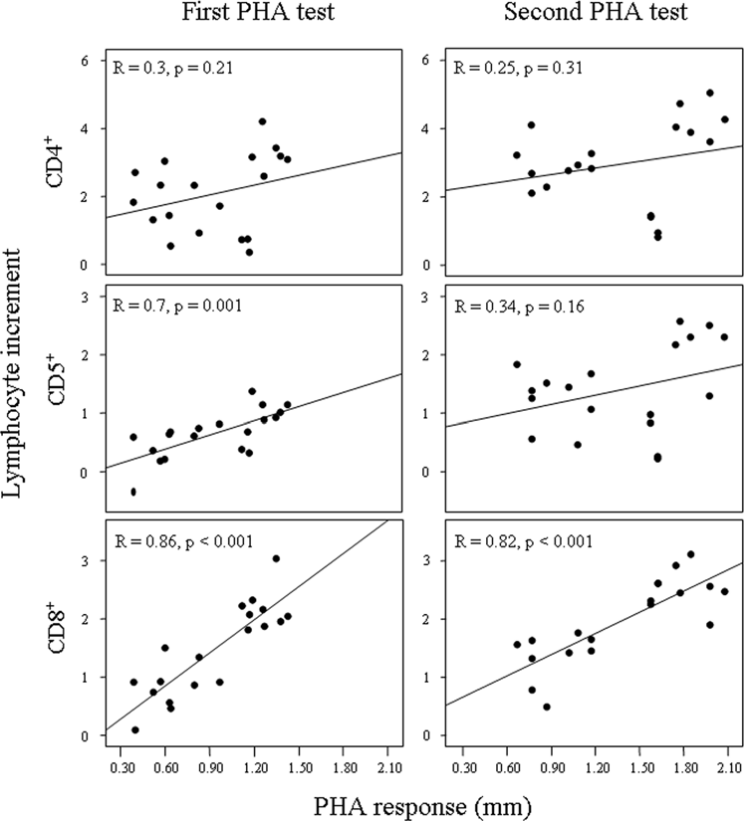
Relationships between local tissue swelling induced by first and second PHA injections and the concomitant changes in circulating lymphocyte subsets. Regression coefficients and statistical significance are shown for each case.

**Figure 4:**
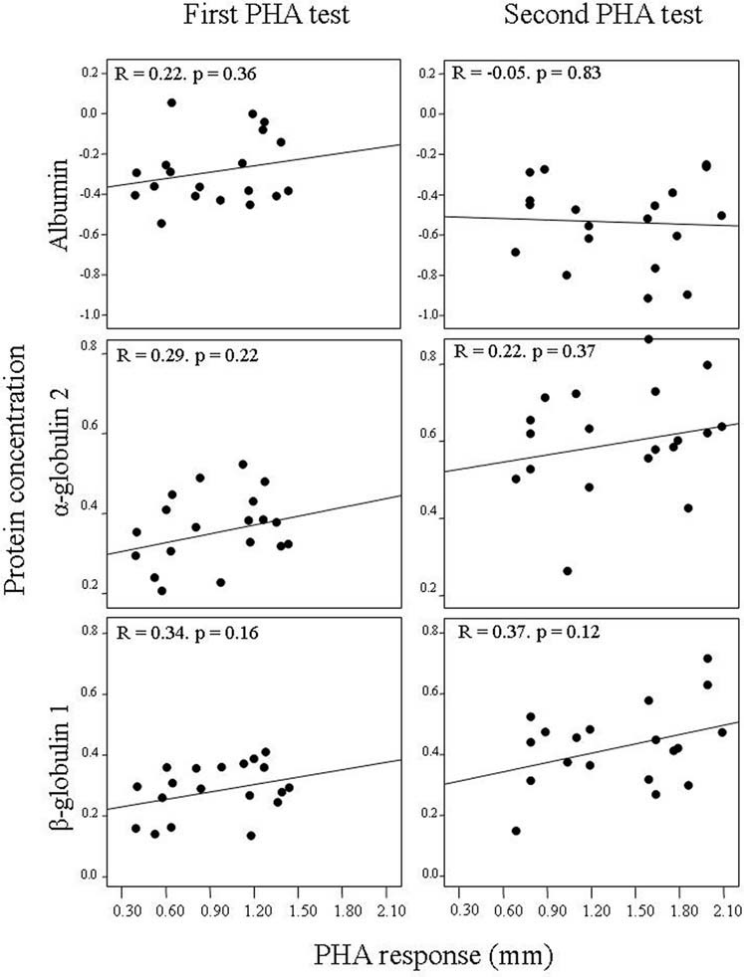
Relationships between local tissue swelling induced by first and second PHA injections and the concomitant changes in circulating protein concentrations (μg/L). Regression coefficient and statistical significance are shown for each case.

Finally, the swelling of tissue at the point of injection was proportional to the proliferation of lymphocytes circulating in blood, the contribution of different types varying between successive exposures (Fig. 3). In the first PHA test, the tissue swelling of experimental birds was positively related to the increase in numbers (from basal levels to 24-h after injection) of the three subsets of circulating lymphocyte types, being significantly correlated for CD5^+^ lymphocytes, as well as those lymphocytes responsible for acquiring a memory response to a novel antigen, CD8^+^ lymphocytes. After a second PHA test the contribution of CD4^+^ and CD5^+^ subsets was moderated, once the organism recognised the antigen from a previous exposure, while maintaining a major role for memory lymphocytes (CD8^+^). Conversely, none of the plasma proteins were significantly correlated to local tissue swelling (r ranges: −0.05–0.37, P-range: 0.12–0.83 for α = 0.01 after Bonferroni correction; see Fig. 4). In multiple regression models, only β-globulins 1 significantly explained variability in tissue swelling (P = 0.007) along with a major contribution from circulating CD8^+^ (P<0.001) as a response to the second PHA injection (model adjusted R^2^ = 0.76, F_2,18_ = 30.19, P<0.001).

The above experiments demonstrate the T-cell mediated nature of the response being measured by the PHA test, through a stronger secondary tissue swelling explained by an increase of circulating lymphocytes which are responsible for acquiring memory from previous antigen exposures. Previous work confirmed an innate component but was unable to identify the contribution of T-lymphocytes to the PHA-induced skin swelling by histology [2], or did not find clear associations between *in vivo* tissue swelling and *in vitro* mitogenic responses to PHA [36]. Here we show that the magnitude of local tissue swelling, while indicating innate immunity to some extent [2], is also reliably reflecting the activation of the T-cell mediated immune system as shown by changes in the bloodstream of T-lymphocyte subsets. The second experiment also suggested that albumin, which can only be obtained through food, may be diverted towards production of acute phase proteins (α- and β-globulins) involved in the non-specific inflammatory process [27]. This could explain why protein-supplemented diets enhance PHA-induced tissue swellings [31], and why the classic positive relationship between these responses and nutritional condition of birds often disappears in captivity conditions [see review also in 31], where standard food is provided *ad libitum*, and thus variability in the access to proteins among individuals is greatly reduced. Therefore, a larger relative contribution of non-specific inflammation is expected in wild populations than we have found in captive birds for explaining variability in local skin swellings. This prediction merits further research, and could be tested both in field conditions and in captivity by manipulating the access to dietary proteins.

In conclusion, the PHA-test can be considered a good measure of T-cell mediated immunity in birds and possibly in vertebrates in general, given that they share the main components of the cell-mediated immune system [37]. Our work solves major concerns about the meaning and interpretation of the test [23], and allows its continued widespread use in diverse research disciplines. However, it is important to note that cellular immunity is just one component of the complex immune system in vertebrates [37], and thus the role of acquired T-cell mediated immunity in combating particular parasites and pathogens is, alone, not enough to explain many immunological patterns and processes [38]. Finally, we expect that the use of flow cytometry and protein electrophoresis will help researchers to elucidate the relative contribution of T-cell mediated and non-specific inflammatory components of the PHA-response when food accessibility varies in quality and quantity, as occurs in field conditions. These methods thus far have been overlooked as analytical tools in immunoecology studies and may add insight, for example, to properly ascertain the environmental and genetic components of the response to PHA [16], [39], which is influenced by nutritional condition early in life [40].

## Materials and Methods

### Experiment 1: Assessment of the secondary immune response

We used 125 birds from 39 parrot species (Order Psittaciformes, see Table 1) varying in body size (range: 20–250 g), ecological, and life history traits [41]. Birds were purchased in the pet market, comprising both wild-caught and captive-bred species [42], and were housed indoor in standard cages and provided with commercial parrot food and water *ad libitum*. After a period of two weeks for acclimatisation, birds were injected in the left patagium with 20μl of 5:1 PHA-P (L8754 Sigma-Aldrich) in PBS, following Smits *et al.* [4]. Patagium width was measured at the point of injection (to the nearest 0.01 mm) three times just prior to and 24 h after injection, using a pressure sensitive micrometer (Baxlo Precission). Average measurements were then used given their high repeatability (*r*>0.99 in all the four measurement instances). The primary response was estimated as the difference between the second and the first patagium width. After a randomly selected time elapsed from the first injection (range: 5–250 days), the same protocol was repeated to estimate the secondary response. The right patagium was used this time, to avoid the potential effects of tissue damage in the previously used patagium. Body mass (to the nearest g) was recorded in both cases to control for its positive effect on the PHA-induced skin swelling in both intra- [31] and interspecific studies [35].

**Table 1:** Species and number of individuals used for assessing two consecutive responses to PHA injection.

Species	Body mass (g)	First PHA response (mm)	Second PHA response (mm)	n
*Agapornis canus*	27.75	1.26	1.86	6
*Agapornis fischeri*	43.83	0.35	0.88	4
*Agapornis personatus*	44.98	0.58	0.88	2
*Agapornis taranta*	49.25	0.73	0.99	1
*Ara (Primolius) maracana*	244	1.5	2.97	1
*Aratinga aurea*	80.19	1.26	1.76	5
*Aratinga jendaya*	123.5	1.97	2.49	2
*Aratinga leucophthalmus*	183	1.61	2.59	2
*Aratinga pertinax*	86.02	1.64	1.9	3
*Barnadius zonarius*	133.75	0.45	0.9	2
*Bolborhynchus (Psilopsiagon) aymara*	28.65	0.92	1.34	1
*Cyanoramphus auriceps*	57.8	0.51	1.06	1
*Cyanoramphus novaezelandiae*	64.45	0.45	1.3	3
*Forpus coelestis*	28.88	0.71	1.22	2
*Forpus passerinus*	24.2	0.41	0.99	2
*Melopsittacus undulatus*	45.3	0.24	0.79	4
*Myiopsitta monachus*	107.71	1.52	2	5
*Nandayus nenday*	137.06	1.61	2.23	6
*Neophema elegans*	46.13	0.34	1.02	2
*Neophema pulchella*	40.85	0.22	0.68	1
*Neopsephotus bourkii*	42.65	0.49	1.16	2
*Pionites leucogaster*	137.25	1.89	2.21	2
*Pionites melanocephala*	148.25	2.07	2.63	2
*Platycercus adscitus*	98.38	0.68	1.03	4
*Platycercus caledonicus*	114	0.59	1.08	1
*Platycercus elegans*	128.67	0.45	0.9	3
*Platycercus eximius*	100	0.54	0.77	4
*Poicephalus senegalus*	126.17	1.54	2.11	3
*Polytelis anthopeplus*	165.14	0.92	1.39	7
*Polytelis swainsonii*	132.75	0.47	0.84	2
*Psephotus haematonotus*	64.94	0.41	0.87	4
*Psittacula krameri*	130.67	1.42	2.08	9
*Pyrrhura cruentata*	101.04	1.08	1.65	4
*Pyrrhura lepida*	71.18	0.93	1.81	2
*Pyrrhura melanura*	75.7	1.1	1.86	3
*Pyrrhura molinae*	66.54	1.26	1.73	7
*Pyrrhura perlata*	78.08	0.78	1.31	4
*Pyrrhura picta*	72.75	1.02	1.67	5
*Pyrrhura rodocephala*	103.98	1.64	2.29	2

### Experiment 2: Circulating lymphocyte and globulin components of the primary and secondary immune responses

A subsample of 20 birds was used for assessing changes in lymphocyte and globulin profiles circulating in blood which are related to T-cell mediated and innate immune responses, respectively. These birds corresponded to five species, which showed very different primary responses (range: 0.54–1.30, see Table 2). After the two-weeks acclimatisation period, birds were bled (0.9 ml taken from the jugular vein) for cytometry and electrophoretic analyses. This sample showed the basal profiles. The first PHA response was elicited ten days later, following the above described protocol, and a second blood sample was obtained when measuring skin swelling 24 h after the injection. The third blood sample was obtained in the same way after provoking a secondary immune response ten days later, using the opposite patagium. A sample of six birds from the same species were used as controls; these birds were injected with just 20μl of PBS (thus do not eliciting an immune response; 2) and manipulated in the same way to obtain blood samples at the same time intervals. Body mass was recorded from all birds in the three sampling instances. Blood was kept cool until centrifugation within the following 12 h to separate plasma for protein electrophoresis.

**Table 2:** Species and number of individuals used to quantify changes in lymphocyte subsets and plasma proteins associated with the primary and secondary responses to PHA injection.

	Species	First response (mm)	Second response (mm)	n
Experimental individuals (injected with PHA)	*Platycercus adscitus*	0.68	1.03	4
	*Platycercus caledonicus*	0.59	1.08	2
	*Platycercus eximius*	0.54	0.77	4
	*Polytelis anthopeplus*	1.06	1.60	4
	*Psittacula krameri*	1.30	1.89	6
Control individuals (injected with PBS)	*Platycercus adscitus*	0	0.04	1
	*Platycercus eximius*	0	0	2
	*Polytelis anthopeplus*	0.01	0.01	3

### Peripheral lymphocyte isolation, flow cytometry and cell sorting

Lymphocytes of peripheral blood were isolated as described previously [43], [44]. Lymphocytes were isolated with the mononuclear cell layer from an aliquot of the blood layered on top of 3ml of Histopaque 1119 (Sigma) and 3ml of Histopaque 1077 (Sigma) and centrifuged at 700g for 30 min. The mononuclear cell layer (above 1077) was collected and cells washed twice with 10ml of C-RPMI [43]. For cell sorting, T-cell characterisation, and analysis of the dynamics of γδ T-cell subsets, fluorescing isothiocyanate-labeled mouse anti-avian CD4^+^ and CD5^+^ antibodies, an R-phycoerythrin-labeled mouse anti-avian CD8^+^α monoclonal antibody (all from Abd-Serotec, Oxford, UK), were used. All antibody concentrations and dilutions were tested prior to starting the animal experiment. For every test, 0.5ml of whole blood were incubated in parallel with the appropriate monoclonal antibodies (30 min in the dark). Aliquots of 10μl were directly analysed using a Guava EasyCyte Plus (Guava Technologies, Hayward, California, USA), to measure absolute numbers of each of the marked subsets. Flow cytometry is nowadays widely recognised as the most accurate method to measure cellular components of immunocompetence in vertebrates, including humans [45]–[49].

### Plasma protein electrophoresis

Total plasma proteins were quantified by the Biuret method [50]. Then, plasma protein fractions were determined on commercial agarose gels (Hydragel Protein (E), Sebia Hispania S.A., Barcelona, Spain) using a semi-automated Hydrasys System (Sebia Hispania S.A., Barcelona, Spain) with manufacturer's reagents to determine the concentration of albumin and globulins (α, β and γ-globulins).

### Statistical analyses

Generalized linear mixed models (GLMM) were used to test for individual changes between successive immune challenges. Contrary to simple matched-pairs tests, a GLMM (with normal error distribution and identity link function) allowed the testing of the effect of the second PHA injection (fixed factor) on skin-swelling responses while simultaneously controlling for initial body mass (continuous variable) and individual identity nested on species (random factor). A second GLMM was used to assess whether the magnitude of individual changes between the two successive PHA-induced swellings was related to individual changes in body mass and time elapsed between injections (as covariates) while controlling for species effects (random factor). Repeated measurement tests were used to examine individual changes in lymphocyte subsets and protein concentrations between the three sampling instances of experiment 2. Sphericity tests indicated the use of the F statistic for our data set. Bonferroni's adjustment to α = 0.01 was applied for analyses of proteins since they were expressed as interrelated concentrations. Simple and multivariate regression models were built to assess the single and combined contribution of changes in lymphocyte levels and protein concentrations on local skin swellings. All tests were performed with SAS v. 8.2 (SAS Institute Inc. 2004).

The first author (JLT) obtained the Spanish certificates that legally allow us to design (Certificate A) and conduct experimental research work using live animals (Certificate B), and all work was done under institutional approval of the competent Spanish wildlife agency (Consejeria de Medio Ambiente, Junta de Andalucía).

## References

[1] Sheldon BC, Verhulst S (1996). Ecological immunology: costly parasite defences and trade-offs in evolutionary ecology.. Trends Ecol Evol.

[2] Martin LB, Han P, Lewittes J, Kuhlman JR, Klasing KC (2006). Phytohemagglutinin-induced skin swelling in birds: histological support for a classic immunoecological technique.. Funct Ecol.

[3] Stadecker M, Lukic M, Dvorak A, Leskowitz S (1977). The cutaneous basophil response to phytohemagglutinin in chickens.. J Immunol.

[4] Smits JE, Bortolotti GR, Tella JL (1999). Simplifying the phytohemagglutinin skin-testing technique in studies of avian immunocompetence.. Funct Ecol.

[5] Clotfelder ED, Ardia DR, McGraw KJ (2007). Red fish, blue fish: trade-offs between pigmentation and immunity in *Betta splendens*.. Behav Ecol.

[6] Amo L, López P, Martin J (2007). Habitat deterioration affects body condition of lizards: A behavioral approach with *Iberolacerta cyreni* lizards inhabiting ski resorts.. Biol Conserv.

[7] Lehmer EM, Clay CA, Wilson E, Jeor SS, Dearing MD (2007). Differential resource allocation in deer mice exposed to sin nombre virus.. Physiol Biochem Zool.

[8] Gervasi SS, Foufopoulos J (2008). Costs of plasticity: responses to desiccation decrease post-metamorphic immune function in a pond-breeding amphibian.. Funct Ecol.

[9] Bize P, Jeanneret C, Klopfenstein A (2008). What makes a host profitable? Parasites balance host nutritive resources against immunity.. Am Nat.

[10] Blas J, Perez-Rodriguez L, Bortolotti GR, Viñuela J, Marchant TA (2006). Testosterone increases bioavailability of carotenoids: Insights into the honesty of sexual signaling.. Proc Natl Acad Sci USA.

[11] Hawley DM, Jennelle CS, Sydenstricker KV (2007). Pathogen resistance and immunocompetence covary with social status in house finches (*Carpodacus mexicanus*).. Funct Ecol.

[12] Laiolo P, Serrano D, Tella JL, Carrete M, Lopez G (2007). Effects of pox-virus infection on the distress calls of lesser short-toed lark *Calandrella rufescens*.. Behav Ecol.

[13] Spottiswoode CN (2008). Cooperative breeding and immunity: a comparative study of PHA response in African birds.. Behav Ecol Sociobiol.

[14] Alonso-Alvarez C, Bertrand S, Faivre B, Chastel O, Sorci G (2007). Testosterone and oxidative stress: the oxidation handicap hypothesis.. Proc R Soc Lond B.

[15] Horak P, Saks L, Zilmer M, Karu U, Zilmer K (2007). Do dietary antioxidants alleviate the cost of immune activation? An experiment with greenfinches.. Am Nat.

[16] Roulin A, Christe P, Dijkstra C (2007). Origin-related, environmental, sex, and age determinants of immunocompetence, susceptibility to ectoparasites, and disease symptoms in the barn owl.. Biol J Linn Soc.

[17] Navarro J, Gonzalez-Solis J (2007). Experimental increase of flying costs in a pelagic seabird: effects on foraging strategies, nutritional state and chick condition.. Oecologia.

[18] Baos R, Jovani R, Forero MG, Tella JL, Gomez G (2006). Relationships between T-cell mediated immune response and Pb, Zn, Cu, Cd and As concentrations in nestling white storks (*Ciconia ciconia*) and black kites (*Milvus migrans*) from Doñana after the Aznalcóllar toxic spill.. Environ Toxicol Chem.

[19] Franson JC, Hoffman DJ, Wells-Berlin A (2007). Effects of dietary selenium on tissue concentrations, pathology, oxidative stress, and immune function in common eiders (*Somateria mollissima*).. J Toxicol Environ Health A.

[20] Hueza IM, Latorre AO, Raspantini PCF, Raspantini LER, Mariano-Souza DP (2007). Effect of Senna occidentalis seeds on immunity in broiler chickens.. J Vet Med A.

[21] Hale KA, Briskie JV (2007). Decreased immunocompetence in a severely bottlenecked population of an endemic New Zealand bird.. Anim Conserv.

[22] Goto N, Kodama H, Okada K, Fujimoto Y (1978). Suppression of phytohemagglutinin skin response in thymectomized chickens.. Poultry Sci.

[23] Kennedy MW, Nager R (2006). The perils and prospects of using phytohaematogglutinin in evolutionary ecology.. Trends Ecol Evol.

[24] Elgert KD (1996). Immnunology.

[25] Vila JM, Calvo J, Places L, Padilla O, Arman M (2001). Role of two conserved cytoplasmic threonine residues (T410 and T412) in CD5 signalling.. J Immunol.

[26] Cho BK, Lian KC, Lee P, Brunmark A, McKinley C (2001). Differences in antigen recognition and cytolitic activity of CD8+ and CD8− cells that express the same antigen-specific receptor.. Proc N Acad Sci USA.

[27] Kaneko JJ, Kaneko JJ (1997). Serum proteins and dysproteinemias.. Clinical biochemistry of domestic animals.

[28] Gentry PA, Loeb WF, Quimby FW (1999). Acute phase proteins.. The clinical chemistry of laboratory animals. 2^nd^ ed..

[29] Nguyen HT, Loeb WF, Quimby FW (1999). Transport proteins.. The clinical chemistry of laboratory animals. 2^nd^ ed..

[30] Cray C, Tatum LM (1998). Applications of avian electrophoresis in avian diagnostics.. J Avian Med Surg.

[31] Alonso-Alvarez C, Tella JL (2001). Effects of experimental food restriction and body-mass changes on the avian T-cell mediated immune response.. Can J Zool.

[32] Reid JM, Arcese P, Keller LF (2003). Inbreeding effects on immune response in free-living song sparrows (*Melospiza melodia*).. Proc R Soc Lond B.

[33] Horak P, Saks L, Ots I, Kollist H (2002). Repeatability of condition indices in captive greenfinches (*Carduelis chloris*). Can J Zool.

[34] Johnsen A, Andersen V, Sunding C, Lifjeld JT (2000). Female bluethroats enhance offspring immunocompetence through extra-pair copulations.. Nature.

[35] Tella JL, Scheurlein A, Ricklefs RE (2002). Is cell-mediated immunity related to the evolution of life-history strategies in birds?. Proc R Soc Lond B.

[36] Bayyari G, Huff W, Rath N, Balog JM, Newberry LA (1997). Effect of the genetic selection of turkeys for increased body weight and egg production on immune and physiological responses.. Poultry Sci.

[37] Tizard I (2000). An introduction to Veterinary Immunology. 6th Ed..

[38] Owen JP, Clayton DH (2007). Where are the parasites in the PHA response?. Trends Ecol Evol.

[39] Pitala N, Gustafsson L, Sendecka J (2007). Nestling immune response to phytohaemagglutinin is not heritable in collared flycatchers.. Biol Lett.

[40] Tella JL, Bortolotti GR, Forero MG, Dawson RD (2000). Environmental and genetic variation in T-cell mediated immune response of fledgling American kestrels.. Oecologia.

[41] del Hoyo J, Elliot A, Sargatal J (1997). Handbook of the Birds of the World. Vol. 4.

[42] Carrete M, Tella JL (2008). Wild-bird trade and exotic invasions: a new link of conservation concern?. Front Ecol Environ.

[43] Finkelstein M, Grasman KA, Croll DA, Tershy B, Smith DR (2003). Inmune function of cryopreserved avian peripheral white blood cells: potential biomarkers of contaminant effects in wild birds.. Arch Environ Contam Toxicol.

[44] Lavoie ET, Grasman KA (2005). Isolation, criopreservation, and mitogenesis of peripheral blood lymphocytes from chickens (*Gallus gallus*) and wild herring gulls (*Larus argentatus*).. Arch Environ Contam Toxicol.

[45] Cooper MD (2002). Exploring lymphocyte differenciation pathways.. Inmunol Rev.

[46] Milston RH, Vella AT, Crippen TL, Fitzpatrick MS, Leong JAC (2003). In vitro detection of functional inmunocompetence in juvenile Chinook salmon (*Oncorhynchus tshawytscha*) using flow cytometry.. Fish&Shellfish Immunol.

[47] Vance CK, Kennedy-Stoskopf S, Obringer AR, Roth TL (2004). Comparative studies of mitogen- and antigen-induced lymphocyte proliferation in four captive rhinoceros species.. J Zoo Wild Med.

[48] Keller JM, Mc Clelland-Green PD, Lee AM, Arendt MD, Maier PP (2005). Mitogen-induced lymphocyte proliferation in loggerhead sea turtles: comparison of methods and effects of gender, plasma testosterone concentration, and body condition on immunity.. Vet Immunol Inmunopathol.

[49] Uchiyama R, Moritomo T, Kai O, Uwatoko K, Inoue Y, Nakanishi T (2005). Counting absolute number of lymphocytes in quail whole blood by flow citometry.. J Vet Med Sci.

[50] Lumeij JT, Mc Lean B (1994). Total protein determination in pigeon plasma and serum: comparison of refractometric methods with the Biuret method.. J Avian Med Surg.

