# Expression of Concern: The PHA-Skin Test Reflects Acquired T-Cell Mediated Immunocompetence in Birds

**DOI:** 10.1371/journal.pone.0049617

**Published:** 2012-11-08

**Authors:** The PLOS ONE Editors

The authors and the editors are issuing this Expression of Concern on the *PLOS ONE* article entitled “The PHA-skin test reflects acquired T-cell mediated immunocompetence in birds”, to make readers aware of concerns over part of the results and conclusions of the study.

The study consisted of two experiments. Experiment #1 was entirely conducted by José L. Tella (JLT) and Martina Carrete (MC), who performed the experimental assays, recorded data and conducted all statistical analyses. Results showed that the PHA test produces a larger secondary than primary immune response, suggesting an acquired immunocompetence in such a response. Experiment # 2 was aimed to assess to what extent the tissue swelling elicited by the PHA test was related to changes in cell profiles circulating in the bloodstream. Experimental assays were again conducted by JLT and MC, who also bled the birds and sent plasma samples to Dr. Lemus for laboratory analyses. Dr. Lemus did not have any contact with the birds used in this experiment and conducted the laboratory analyses blindly (the plasma samples were labeled with a code). MC crossed the laboratory results provided by Dr. Lemus with the tissue swelling measurements, body masses and identity of birds obtained by JLT and MC, which were unknown to Dr. Lemus, and performed the statistical analyses. These analyses showed highly significant correlations between individual tissue swelling and circulating CD5^+^ and CD8^+^ lymphocyte subsets (Figure 3), thus supporting the conclusions of experiment #1 on the use of the PHA test as an indicator of acquired T-cell mediated immunocompetence in birds.

In January 2012, the Ethics Committee of the Spanish Superior Council of Scientific Research (CSIC) initiated a formal investigation in relation to concerns about potential scientific misconduct by Dr. Lemus. The investigation was recently completed and in relation to this article, the Ethics Committee of CSIC was not able to clarify in which external laboratory Dr. Lemus conducted the flow cytometry analyses. The fact that the analyses were carried out blindly makes it unlikely for the significant correlations between tissue swelling and circulating CD5^+^ and CD8^+^ lymphocyte subsets shown in Figure 3 to be the result of data fabrication. The published correlation coefficients significantly differ from those obtained by chance, being near twice the upper confidence intervals for coefficients of correlations obtained from 100 randomizations of tissue swelling data. However, the uncertainty about the laboratory where the cytometry analyses were performed does raise concerns about the reliability of the results.

The validity of the results and conclusions derived from Experiment # 1 has not been questioned. However, in light of the outcome of the institutional investigation, we want to make readers aware that concerns remain in relation to the cytometry analyses included in Experiment # 2.

The authors sincerely apologize for all inconvenience to the *PLOS ONE* readership.

